# Enhanced Wetting and Adhesive Properties by Atmospheric Pressure Plasma Surface Treatment Methods and Investigation Processes on the Influencing Parameters on HIPS Polymer

**DOI:** 10.3390/polym13060901

**Published:** 2021-03-15

**Authors:** Miklós Berczeli, Zoltán Weltsch

**Affiliations:** Department of Innovative Vehicles and Materials, GAMF Faculty of Mechanical Engineering and Computer Science, John von Neumann University, Izsáki St. 10, 6000 Kecskemét, Hungary; berczeli.miklos@gamf.uni-neumann.hu

**Keywords:** polymer, wettability, plasma, surface treatment, adhesion, adhesive

## Abstract

The development of bonding technology and coating technologies require the use of modern materials and topologies for the demanding effect and modification of their wetting properties. For the industry, a process modification process that can be integrated into a process is the atmospheric pressure of air operation plasma surface treatment. This can be classified and evaluated based on the wettability, which has a significant impact on the adhesive force. The aim is to improve the wetting properties and to find the relationship between plasma treatment parameters, wetting, and adhesion. High Impact PolyStyrene (HIPS) was used as an experimental material, and then the plasma treatment can be treated with various adjustable parameters. The effect of plasma parameters on surface roughness, wetting contact angle, and using Fowkes theory of the surface energy have been investigated. Seven different plasma jet treatment distances were tested, combined with 5 scan speeds. Samples with the best plasma parameters were prepared from 25 mm × 25 mm overlapping adhesive joints using acrylic/cyanoacrylate. The possibility of creating a completely hydrophilic surface was achieved, where the untreated wetting edge angle decreased from 88.2° to 0° for distilled water and from 62.7° to 0° in the case of ethylene glycol. The bonding strength of High Impact PolyStyrene was increased by plasma treatment by 297%.

## 1. Introduction

Different industries have developed very rapidly in the past decades, where it is important that the surface of the materials used have surface characteristics like coatings, adhesive technologies, painting applications, soldering, and brazing [1,2]. Examples of such properties include hardness, corrosion resistance, and wear resistance [3]. Interface properties play a major role in the life of component and material pairing, as most damage processes start on the surface, and the surface is usually exposed to external damages during the different weather conditions and mechanical stresses [4,5]. Further interface property may be the wetting ability of a surface [6]. It may be necessary to have a surface that repels liquids such as car windscreens with rain, oil impregnating of mechanical parts, or a surface that has good wetting property [7,8,9]. A good wetting property means that a liquid can spread fully on the surface [10,11,12]. An example of the need for a good wetting property is for the adhesion bonding techniques [13,14]. If the wetting behaviour of the surface is bad, then it will result in a poor bonding strength and poor joining quality [15]. Examples of such bonding technologies are coating, gluing, and soldering. Interface properties can be modified by different conventional and advanced surface treatment procedures [7,15].

There are several technologies for treating surfaces, which can be divided into two groups, such as conventional surface treatments and modern surface treatment processes. Surface treatments can be, for example, surface workouts or thermochemical surface treatments, and modern surface treatments can be, for example, physical vapour deposition (PVD), chemical vapour deposition (CVD), ion implantation, plasma surface modification, and laser beam surface modification [16,17,18]. These surface treatments may be suitable for changing wetting properties [19]. There are two innovative surface treatments especially for polymer materials that can enhance wetting behaviour, these are the laser beam and plasma surface treatments [7,20]. In the present research, in order to broaden and expand the range of applications of polymers, in order to advance their final development, we currently highlight the plasma surface treatment on the polymer base material.

**Figure 1:**
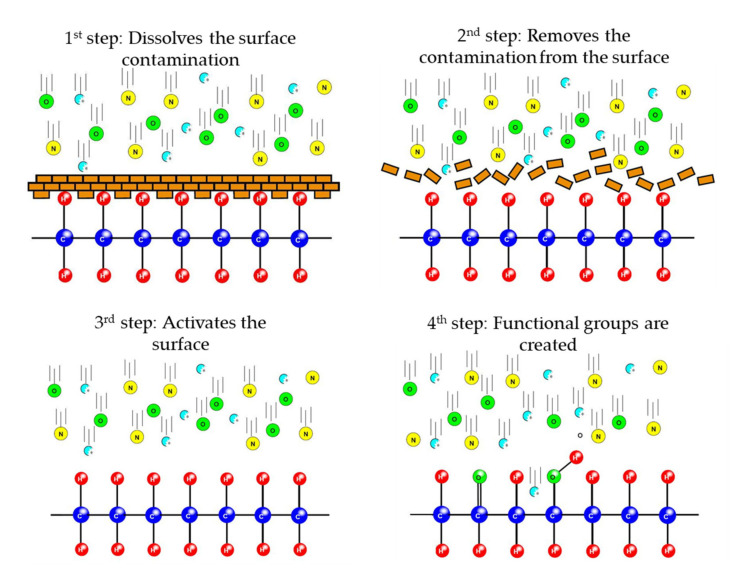
The 4 main process steps of the cleaning and activating effect of plasma jet surface coating [22].

Two types of plasma are used in the industry for machining, high temperature (hot) and low temperature (cold) plasma [21]. The hot plasma is high in temperature since the plasma-forming elements are in a thermal equilibrium, so the energy of the ions, electrons, and neutral particles in the plasma is nearly the same. Thermal plasmas, for example, an argon gas plasma, can reach temperatures of 11,200 K. Further, thermal plasmas are characterized by the fact that the plasma-forming elements have a Maxwell velocity distribution or particle velocity, which means that the particles are not in continuous interaction with each other but move freely while sometimes they collide with each other [22]. For application, hot plasma is used for cutting, hazardous waste processing, coating, and spraying [23]. However, non-thermal, i.e., cold plasmas, are not in thermodynamic equilibrium because the particles contained therein do not have the same temperature and have a velocity distribution that does not correspond to the velocity distribution defined and described in the Maxwell–Boltzmann distribution [24]. In non-thermal plasmas, the ions and the neutral particles have a low temperature while the electrons have high temperatures. This, however, does not lead to a high-temperature increase, as the electrons have low density and low thermal capacity. Due to their low temperature, cold plasma is widely used in the industry. Non-thermal atmospheric plasmas are used for surface cleaning, surface activation, surface etching, and coating [25]. During these processes, two surface reactions occur primarily. One surface reaction is a physical reaction that results from ionic activation. The other is the free radical-induced chemical reaction. During the physical reaction, the particles in the plasma are ionized to give charge and kinetic energy. As these particles collide with the surface, they remove some of the atoms and molecules from the surface [26]. This bombardment increases the molecular surface roughness and facilitates the adhesion of the interface [27]. During the chemical reaction, chemically active free radicals are formed on the surface, which increases the chemical reaction potential of the surface. The efficacy of the treatment may depend on many parameters, such as plasma power density or plasma gas mixture [22]. The steps of the surface treatment are shown in Figure 1.

In similar research, the contact angle values they have been able to achieve are quite varied according to our goal. Polycarbonate polymer was surface treated with Diffuse Coplanar Surface Barrier Discharge (DCSBD) atmospheric pressure plasma to reduce the wetting angles from around 70° to around 30° for water. The treatment parameter was the duration of treatment, and if the sample was treated 5 s, the wetting contact angle values decreased, but if the contact angle values were treated for 10 s, they started to increase again [28]. In another study, poly (methyl methacrylate) was treated with a DCSBD electrode atmospheric pressure plasma system, the distance between the treated surface and DCSBD was 0.3 mm, in which the water contact angle values decreased from around 85° to 50° with a treatment time of 3 s while using only 1 s led to a decrease from 78.4° to 40.1°. They created a higher roughness on the surface of treated samples, 6.90 nm compared to the 1.25 nm of the untreated surface [29]. Even in this research, if further treated, the wetting values deteriorated. In a study of atmospheric pressure plasma-treated polyethylene and polypropylene, as a result of the treatments, the surface of both raw materials became rougher. This may be because they treated their surface for a longer period of time, between 15 and 90 s, and used a Ni-Cr one-electrode plasma device with argon gas flow to induced the plasma jet. Furthermore, the treatment distance was 2 mm which creates a limit of usage and causes high damage on the surface [30]. There is little literature that has examined the increase in adhesive strength as a result of plasma surface treatment. Glass was bonded after a source of diffuse, atmospheric pressure plasma was used with string electronegative gases like pure oxygen without any admixture of helium or argon. The plasma is generated in a thin 0.5 mm thick layer suitable for flat materials like glass and polymers, especially for foils. The samples were positioned 0.3 mm above the surface of the glass and treated for 5 s and the result of the treatment there was a 45% improvement in bond strength compared to the untreated joint’s adhesive strength [31].

A scientific shortcoming in the surface treatment of modern base materials has been identified. The aim of this research is to map the technological parameters and the effect of the plasma jet surface treatment on the changing effect of the wetting phenomena and the strength of the adhesive joints. To assess this information, wettability changes, direct measurement of wetting edge angle and surface free energy calculation, measurement of substantive characteristics over dissolved surfaces, and overlapping bonded bonding with well-wetted surfaces should be performed and measured at different scanning speeds of the plasma head and plasma head spacing.

## 2. Materials and Methods

### 2.1. HIPS Polymer

High Impact PolyStyrene (HIPS) was used as a substrate, manufactured by A-Plast. Polystyrene is one of the most widely produced and used plastics in today’s vehicle industry. Impact-resistant polystyrene is inexpensive, easily thermoformed, and easy to produce with good mechanical properties. Due to its easy thermoformability, many different shaped objects can be made from it. Pure polystyrene is an amorphous, transparent material with a softening point of about 100 °C, it is brittle at room temperature, micro-cracks could appear in a short time due to internal stress, (this reason is why the plasma surface activation is a good method for these type of polymers), and it dissolves well in most organic solvents. Its short-term mechanical properties are good, but it is sensitive to stress corrosion. The thickness of the used HIPS base material is 5 mm, one side of which is provided with a protective film that can be torn off before usage, this side was used for the experiments. The protective film ensured that a homogeneous and clean, intact surface was always treated, and the surface roughness was the same on the side of the protective film. The samples were cut with hydraulic plate shears with a final size of 25 mm × 55 mm × 5 mm. The 25 mm width and 55 mm length were set to test the bonding technology. The surfaces were protected from all kinds of thermal effects. Due to the protective film, no alcohol cleaning was performed.

### 2.2. Plasma Surface Treatment

For the surface treatment, the PlasmaTreat OpenAir^®®^ system has been used, which is able to modify the surface at atmospheric pressure without introducing a large amount of heat into the surface. The special feature of the device is that it cleans and activates the surface at the same time. Plasma was generated by an FG5001 plasma generator with a maximum output power of 1000 VA and an output frequency of 19–20 kHz. An RD1004 type plasma head and an HTR12 high voltage transformer were connected to the generator. The machine was operated at 21 kHz during the experiment. The plasma emitting head was mounted on an ABB (Zürich, Elveția) robot arm and the parameters were controlled by the robot.

First, the test parameter set was determined for HIPS plasma surface treatment. The performance of the plasma equipment was set to maximum. Two parameters were changed in this research, the plasma surface treatment distance and treatment speed. The plasma surface treatment distance is measured between the plasma head and the surface of the substrate whose were parallel in the setup. The minimum distance between the plasma head and the surface of the HIPS was 4 mm, because using 3 mm damaged and degraded HIPS, while the aim of the research was not to alter the surface aesthetically. The maximum distance was 25 mm. The next step was to examine the speed at which no visible change occurred on the surface. From this point of view, a speed of 0.7 m/min was chosen as the slowest treatment speed, as below this speed the surface of the plate is damaged and its subsequent industrial applicability is not advantageous. The fastest speed applied to the surface treatment was 24 m/min.

The plasma surface activation path was made with a single, one-way scanning on the surface with a rotary plasma surface jet, with a rotation speed of 2000 rpm. Using this method the surface activation was homogenous on the 25 mm wide specimens. In the case of plasma-treated samples, seven plates were treated with each technological parameter variation, and then the determination of the interfacial conditions was measured with distilled water and ethylene glycol. The wetting measurements thus obtained were also evaluated four times. During the testing of the plasma technological parameters on HIPS, the treatments were performed with 7 different treatment distances and 5 speed values, 35 combinations with the two liquid were followed by the measurements.

### 2.3. Wettability Measurement

In the next step, the plasma surface treatment contact angle measurement was performed on the specimens. During the contact angle measurement process, the drop does not fall on the surface but is just applied to the surface, which is good because it gives an idea of the wetting of the surface and not how the surface behaves against a drop falling on it. The basis of this method is that there are a camera and a light source in line with the drop. The camera is facing the light source with drops between them. A micropipette system was used for the droplet, using 5 µL of distilled water and ethylene glycol drops for the measurement. For dripping, two liquids had to be used for Fowkes’ method of calculating interfacial energy. The Fowkes’ method Equation (1):

(σ_L_^D^)^(1/2)^*(σ_S_^D^)^(1/2)^+(σ_L_^P^)^(1/2)^*(σ_S_^P^)^(1/2)^ = ((σ_L_)*(cosθ+1))/2, (1)

θ = the measured wetting contact angle on one liquid droplet,

σ_L_^D^ = dispersive component of the surface tension of the wetting liquid,

σ_L_^P^ = polar component of the surface tension of the wetting liquid,

σ_L_= σ_L_^P^+ σ_L_^D^,

σ_S_^D^ = dispersive component of the surface energy of the solid,

and σ_S_^P^ = polar component of the surface energy of the solid.

One liquid is distilled water and the other is ethylene glycol in this paper. The droplets were performed at a temperature of 20 °C ± 1 °C, as the polar and disperse components of the surface tension of the water used and the ethylene glycol are known at this temperature. Humidity ranged from 40 to 60%, which is adequate. The polar and dispersed components of distilled water at 20 °C were 51.0 mN/m and 21.8 mN/m, and the polar and dispersed component of ethylene glycol at 20 °C was 19 mN/m and 29 mN/m. Using the two different liquids and measured on the same modified surface on HIPS, the actual surface energy can be calculated using the Fowkes Equation (1) as a simultaneous equation. Prior to the wetting measurements, the wetting conditions of the HIPS had to be assessed with two different liquids to be used, in order to determine the wetting contact angles of the untreated pieces and the initial interface energy values. A total of 2-2 drops of water and ethylene glycol were made on 10 untreated plates in order to determine the wetting conditions of the untreated polymer surface.

Summarizing the wetting measurement recordings, each recording was evaluated twice to obtain an angle value of 80. Plasma surface treatment loses its effect after some time, so a time dependence test was performed with the optimal treatment parameters, thus simulating the environmental load of the workpiece after treatment in industrial use. For the study, wetting measurements were performed only with distilled water. Samples were stored in a dark, completely enclosed space at 25 °C ± 2 °C after plasma surface treatment.

### 2.4. Surface Roughness Measurement

Non-contact surface roughness was performed on treated and untreated specimens on a Rodenstock RM600-S instrument, for the surface roughness measurement non-contact laser micro height measurement was used on the samples. In the geometrical centre of the specimen, 5 surface roughness measurements were performed both parallel (Y direction during the plasma treatment) and perpendicularly (X direction) with the plasma surface treatment. In these directions, the machine scans an 8 mm long line with the limits of ±1 µm with a resolution of 4 nm in this measuring setting and measures the roughness values such as Ra, Rz, and Rmax. As a result, the 5 measurements were averaged and used for discussion both with the untreated and plasma-treated cases.

### 2.5. Adhesive Breaking Test

For the adhesive test Loctite 4080, a cyanoacrylate/acrylic hybrid structural adhesive was used. It is a two-component, translucent, slightly yellowish adhesive that provides high toughness and excellent adhesion to a variety of materials. During the gluing, 25 mm overlapping doubts were made, the amount of glue was 0.10 g in each case, and the samples were compressed over the entire surface, with the aim of reaching a 0 mm gap. The adhesive was applied to the centre of the 25 mm × 25 mm adhesive area. The samples were then scattered for 24 h. After the determination of the optimal plasma surface treatment technology parameters, the appropriate number of samples were surface treated and bonded with these parameters, which were compared with the untreated surface samples.

## 3. Results and Discussion

### 3.1. Effect of Plasma Treatment on Surface Roughness

**Figure 2:**
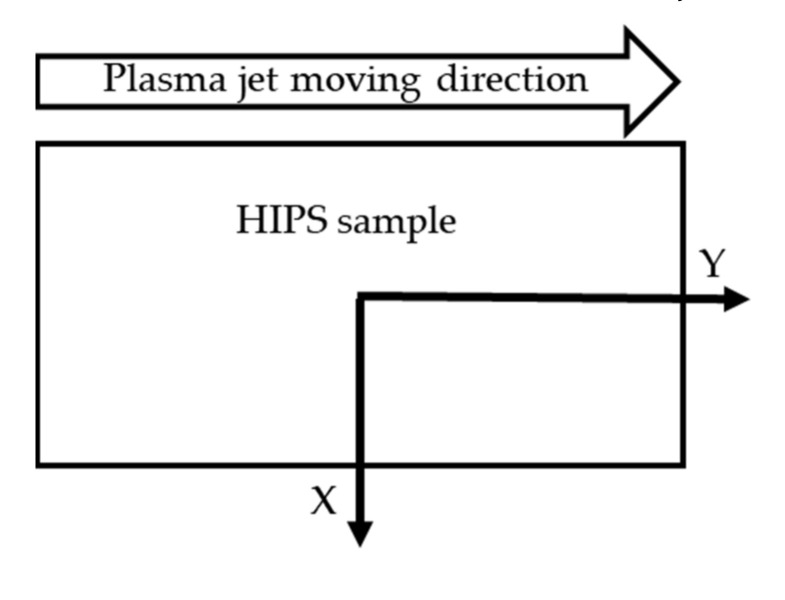
Illustration of the definition of the X direction perpendicular to the plasma gas treatment and the Y direction parallel to the treatment.

The plasma surface treatment reduced the surface roughness values overall compared to the untreated HIPS specimen. The rate of decrease in the Y direction (parallel to the direction of the plasma jet movement) was greater, which may have been due to the direction of travel of the treatment jet (Figure 2).

**Figure 3:**
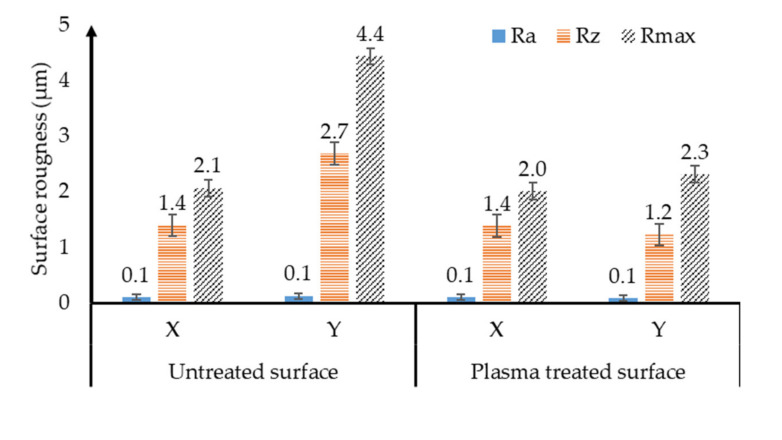
Changes in surface roughness for untreated and plasma-treated polymer test pieces, examining test directions parallel (Y direction) and perpendicular (X direction) to the plasma surface treatment separately, analysing the difference between Ra, Rz, and Rmax values.

Compared to the untreated, in the Y direction, the average surface roughness (Ra) of the treated specimen decreased by 37.5% and the height of roughness (Rz) decreased by 54.3%, this can be seen in Figure 3.

The reason for the change may be that the inequalities resulting from the production of the sample are smoothed out by the plasma treatment. In the X direction, the measured surface roughness indices did not change significantly. There was no significant surface modification in the surface aesthetics.

The specimen SEM investigation showed no significant change in the surface that would be generated by the plasma beam surface treatment. This corresponds to the aim of the research, as the requirement of the industrial and automotive industry actors is to improve the adhesion without changing the roughness quality of the surface.

### 3.2. Wettability and Surface Energy Measurements

According to the wettability measurements the untreated HIPS had 88.2° ± 1.5° contact angle in the case of distilled water, while in the case of ethylene glycol it was 62.7° ± 1.5°, the standard deviation was calculated based on the data set for ethylene glycol. Using the contact angles measurement results on the untreated HIPS plate and the Fowkes formula the surface energy values shown as later in the figures as untreated plates with a horizontal line were calculated, the polar component of the initial surface energy of the untreated polymer was 4.4 mN/m, and the value of the component of the dispersion was 23 mN/m.

**Figure 4:**
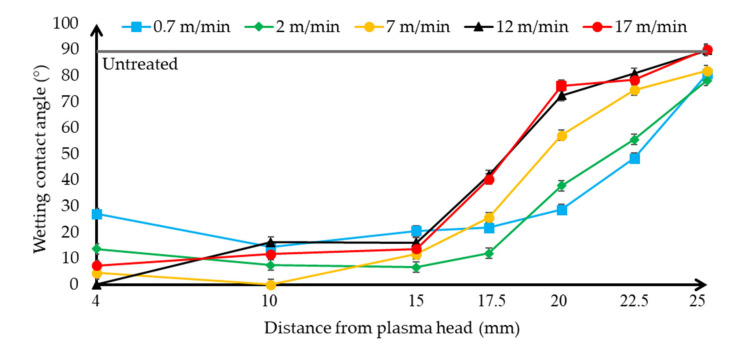
Evolution and tendency of the wetting contact angle of distilled water on High Impact PolyStyrene (HIPS) specimen depending on the distance of the plasma surface treatment head, using different plasma head scanning speeds. The untreated sample’s contact angle using distilled water is shown.

**Figure 5:**
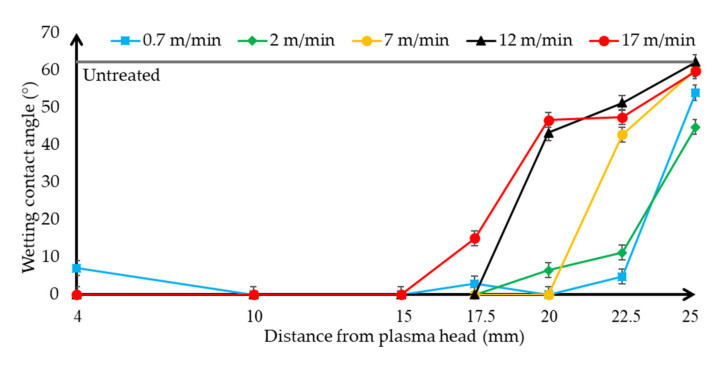
Evolution and tendency of the wetting contact angle of ethylene glycol on HIPS specimen depending on the distance of the plasma surface treatment head, using different plasma head scanning speeds. The untreated sample’s contact angle using ethylene glycol is shown.

The wetting contact angle values of the surface of the treated HIPS polymer samples has been obtained with 5 µl droplet of distilled water (Figure 4) and ethylene glycol (Figure 5), The results were plotted on a separate diagram to discuss the behaviour of the two liquids with different surface energy according to the plasma jet surface treatment distance and the plasma jet as a function of movement speed.

The averages of the values measured on the untreated HIPS polymer’s surface are marked with a grey line on the diagrams. After the plasma surface treatment, the surface of the polymer showed complete hydrophilicity against liquids with several parameters. It can be seen from the trend of the diagrams that compared to untreated samples (the grey line) when the start of the increase of energy of the plasma beam has been performed on the surface, so the plasma jet head slowed down with the scanning speed and/or closing the distance to approach the samples, the wetting contact angle values decreased for both liquids. However, these wettability changes trends according to the plasma jet surface treatment parameters have an optimum because if too much energy is applied to the surface with 4 mm plasma jet distance and 0.7 m/min movement speed, the wetting contact angles are already increasing and visual discoloration can also be seen on the HIPS polymer’s surface. These damage causative values were typically at a distance of 4 mm or below and at a slower speed than 0.7 m/min, these parameter areas should be avoided. The contact angle values of the water started to decrease with the higher energy irradiation, due to the fact that the surface energy of distilled water is 72.9 mN/m, which is higher than the other liquid, so the contact angle decreasing mechanism of action is delayed compared to ethylene glycol. The water from the untreated samples from 88.2° and the ethylene glycol from 62.7° can be obtained by plasma surface treatment to completely spread out and approach the hydrophilic effect, so the treatment efficiency was close to 100% considering the reduction of the wetting contact angles. This trend is predicted by adhesion bonding studies since there is a relationship between interfacial wetting and adhesion. With these parameter sets and wetting contact angle measurement, the nominal value of the surfaces can be easily and efficiently determined and thus quickly optimized for a gluing technology.

**Figure 6:**
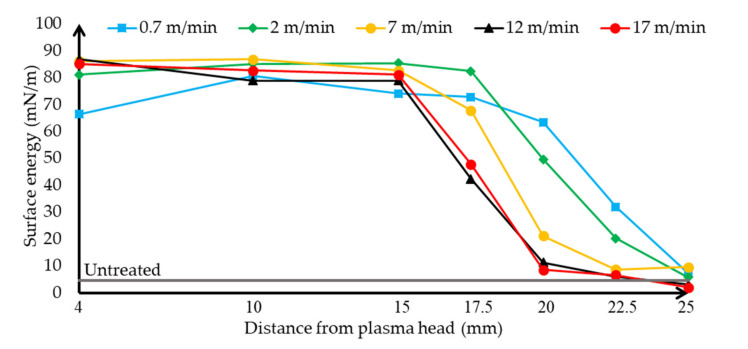
Evolution and tendency of the polar surface energy of distilled water on HIPS specimen depending on the distance of the plasma surface treatment head, using different plasma head scanning speeds. The untreated sample’s contact angle using ethylene glycol is shown.

**Figure 7:**
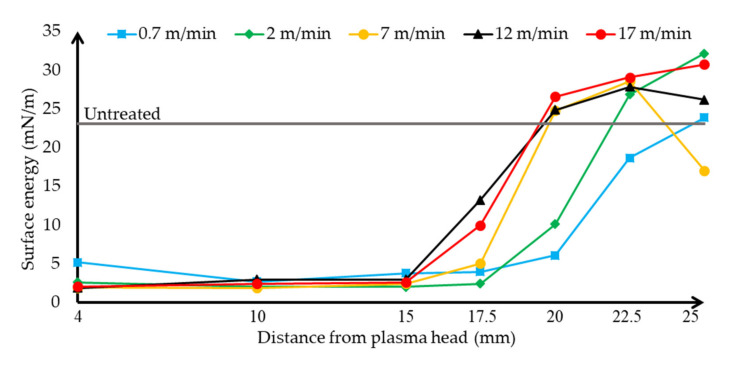
Evolution and tendency of the dispersed surface energy of ethylene glycol on HIPS specimen depending on the distance of the plasma surface treatment head, using different plasma head scanning speeds. The untreated sample’s contact angle using ethylene glycol is shown.

Based on the wetting contact angle values obtained in the previous measurements, the surface energy values on the surface of the treated HIPS plates were calculated by mathematical methods and the Fowkes formula. The polar (Figure 6) and dispersed (Figure 7) interface energy components are plotted separately.

The diagrams show that the components of polar and dispersed energy change in harmony with each other. The changing rate of the dispersed and polar surface energy is correlated with the changing of the contact angles according to Fowkes’ theory of the calculation of the surface energy. When the contact angles were low, the polar component of the interfacial energy of the treated sample increased greatly, while as the effect of the treatment on the phenomena deteriorated, the polar energy component of the surface decreased and the dispersed component increased. This can be explained by the fact that the polar component of the surface is increased by the plasma treatment, as hydroxyl and carboxyl groups are formed on the surface of the plastic. When the surface was treated too slowly and close, these hydroxyl and carboxyl groups were damaged, explaining why there was not as much improvement in the polar component of the surface energy. In addition, when treated too fast, these groups could not form, so the polar component of the surface energy decreased. Thus, in accordance with the measured wetting contact angle values, it can be said that the amount of energy of the polar component of the surface has a positive effect, while the dispersed component has a negative effect on the wetting of the surface and thus on its adhesion.

**Figure 8:**
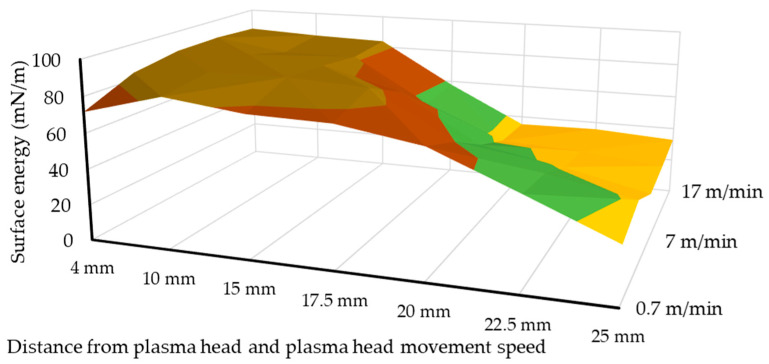
Surface tendency of the summarized surface energy components on HIPS specimen depending on the distance of the plasma surface treatment head and using different plasma head scanning speeds.

In addition, in order to map the plasma surface treatment of polymer products, it is expedient to treat the sum of the polar and disperse components of the surface energy values together in Figure 8, in order to correctly classify the technology.

During the representation of the data, a surface is obtained from the measured values, so it can be seen which plasma beam parameters must be used to achieve the appropriate level of mN/m energy level. By examining the slope and shape of the surface obtained from the parameter set and the wetting result, the optimal surface treatment parameters from the point of view of production and application can be determined. Depending on the technological parameters of the surface shape, it can be stated that the intensity of the irradiation of the plasma on the surface fundamentally influences the improvement of the wetting. However, excessive energy intake reduces the interfacial energies because they damage the surface of the polymer. According to the wettability measurements, the optimal plasma surface treatment parameter with a jet head distance to the surface of the polymer of 4 mm and with a treatment movement speed of 7 m/min has been determined. The time dependence of the surface treatment activity and the gluing tests were performed on the test pieces made with the optimal HIPS plasma surface treatment parameter determined with the parameter resolution detail used in the present research.

Researchers have investigated the effect of wetting change on several different polymers by different plasma surface treatments, but the wetting change result achieved by the atmospheric plasma jet with compressed air is unique in our research and its efficacy can be clearly demonstrated compared to the literature references [32]. In our case, a hydrophilic state was achieved at a significantly higher velocity of movement, i.e., using a low plasma irradiation time. Compared to typical research, the resolution detail of the parameters is also high, and thus the mapping of the interfacial energy.

### 3.3. Time Dependence

**Figure 9:**
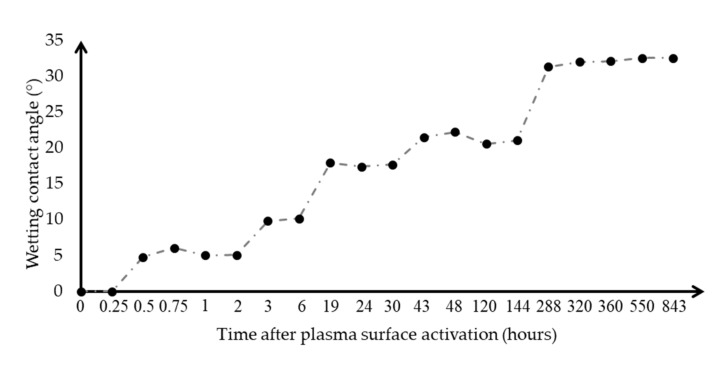
The rate of increase in the wetting contact angle of distilled water over time on a surface-treated HIPS sample at zero time. The dotted line has no physical content, it shows the trend.

The values measured with its elapsed time are shown in Figure 9.

Based on the results, it can be seen that the more time elapsed since the treatment, the more the angle of the water began to increase. This may have been because the free radicals on the surface began to react with the surrounding air, with atoms sitting in the empty spaces. In other words, the surface began to get dirty. The effect of the plasma jet surface treatment fully maintained the hydrophilic behaviour at level 1, for the first 15 min, after 30 the sweep angle was already clearly measurable and the hydrophilic state was eliminated. This changed to level 2 after 3 h and the wetting edge value started to increase more, this level increased to a wetting contact angle of 20° at level 3, this wetting state was maintained for a long time for about 144 h and then at level 4 was reduced. The typical wetting state showed a 30–35° rim angle and persisted up to 843 h (35 days).

### 3.4. Adhesive Breaking Test

**Figure 10:**
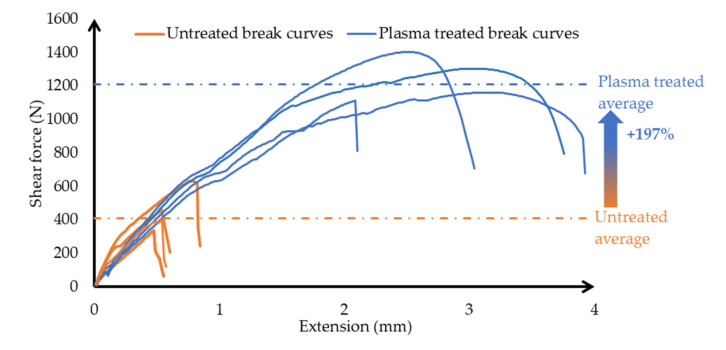
The diagram shows the breaking test of overlapping bonded joints as a function extension, indicating separately the main features of the break curves of untreated and plasma-treated samples. The dotted lines show the average breaking strength of the untreated and treated surface bonded HIPS polymer pairs, and the increment is displayed.

Figure 10 shows the strength results obtained after the breaking test of untreated and plasma-coated surface-treated specimens.

The diagram also shows a dotted line indicating the average shear force of untreated and treated test pieces. It can be seen from the tendency of the load curves that the glued overlapping joints can withstand the load and then the force decreases rapidly and breaks along the gluing. The untreated samples withstood an average tensile strength and shear force of 0.65 MPa (408 N), while due to the change in surface roughness and the increase in interfacial energy due to plasma surface treatment, this tensile strength was increased to 1.94 MPa (1211 N). The treatment resulted in a 297% adhesive strength compared to the untreated surfaces’ HIPS substrate. The standard deviation of the ruptures was 0.224 MPa (140.1 N) for the untreated and 0.13 MPa (82.7 N) for the treated. Furthermore, the detachment of the adhesive during the tearing became more homogeneous on the surface, which is an additional advantage of the treatment. The increase in strength can be clearly and significantly related to the increase in wetting and interface energies. As a result of plasma jet treatment, the activated molecular groups on the interface are able to form a better bond with the acrylic/cyanoacrylate adhesive used.

In comparison with the measurement results found in the literature search, it can be said that in our work we managed to almost triple the strength in a shorter plasma treatment time [33]. This result is unique and testing on HIPS for plasma jet surface treatment has not been analysed.

## 4. Conclusions

Increasing tightening to protect the environment, the emergence of special new materials and their use requires the use of appropriate bonding technology. In order for the bonding quality to be adequate, a surface treatment must be applied to improve the wetting properties. One such surface treatment technology that can change this interfacial phenomenon is atmospheric pressure plasma surface treatment. As a summary of the results using HIPS polymer obtained during the research:

- Based on the surface roughness test, the treated surface became smoother compared to the untreated plate in the direction of treatment. The untreated plate had an average surface roughness of 0.1 µm, while the treated plate had an average surface roughness of 0.1 µm and the unevenness of the untreated sample was 2.7 µm, while the treated sample had a height of 1.2 µm.
- The surface treatment improved the wetting properties of the specimens. For the untreated plate, the water had a wetting contact angle of 88.2° and for ethylene glycol 62.7°, while for the treated pieces it reached 0°, it was hydrophilic for both liquids.
- The optimal plasma treatment distance parameter was 4 mm, but at this distance, if treated too slowly, the wetting contact angle values deteriorate. When the surface of the specimen was treated from greater distances, but just as slowly, we did not achieve such good wetting angle values as if we had treated it up close more quickly. As we moved away and accelerated, the treatment began to lose its effect and there was a case where we got the same wetting contact angle as for untreated plates.
- The optimal treatment parameter is a treatment distance of 4 mm with a treatment speed of 7 m/min.
- The specimens treated with the optimal parameter were connected in an overlapping manner, with which a 296% increase in adhesive strength was achieved compared to the connected untreated plates.

## Data Availability

The data presented in this study are available on request from the corresponding author.
